# Detection of Urine Metabolites in a Rat Model of Chronic Fatigue Syndrome before and after Exercise

**DOI:** 10.1155/2017/8182020

**Published:** 2017-03-22

**Authors:** Changzhuan Shao, Yiming Ren, Zinan Wang, Chenzhe Kang, Hongke Jiang, Aiping Chi

**Affiliations:** ^1^College of Arts and Sciences, Shanghai Maritime University, Shanghai 201306, China; ^2^Laboratory of Nutrition and Hygiene, Shaanxi Normal University, Xi'an 710119, China; ^3^School of Sports, Hebei Normal University, Shijiazhuang 050090, China

## Abstract

*Purpose.* The aim of the present study was to elucidate the metabolic mechanisms associated with chronic fatigue syndrome (CFS) via an analysis of urine metabolites prior to and following exercise in a rat model.* Methods.* A rat model of CFS was established using restraint-stress, forced exercise, and crowded and noisy environments over a period of 4 weeks. Behavioral experiments were conducted in order to evaluate the model. Urine metabolites were analyzed via gas chromatography-mass spectrometry (GC-MS) in combination with multivariate statistical analysis before and after exercise.* Results.* A total of 20 metabolites were detected in CFS rats before and after exercise. Three metabolic pathways (TCA cycle; alanine, aspartate, and glutamate metabolism; steroid hormone biosynthesis) were significantly impacted before and after exercise, while sphingolipid metabolism alone exhibited significant alterations after exercise only.* Conclusion.* In addition to metabolic disturbances involving some energy substances, alterations in steroid hormone biosynthesis and sphingolipid metabolism were detected in CFS rats. Sphingosine and 21-hydroxypregnenolone may be key biomarkers of CFS, potentially offering evidence in support of immune dysfunction and hypothalamic-pituitary-adrenal (HPA) axis hypoactivity in patients with CFS.

## 1. Introduction

Chronic fatigue syndrome (CFS) is a debilitating illness associated with serious changes in physical, mental, and occupational well-being and, frequently, social isolation, with a prevalence of 0.2–2.6% and 1.9–3% in Western countries and China, respectively [1–5]. The typical presentation of CFS is characterized by sudden onset of a flu-like illness in which the predominant symptom is severe and lasting fatigue that greatly reduces activity [6].

Several hypotheses have been proposed for the pathogenesis of CFS including oxidative stress, hypothalamic-pituitary-adrenal (HPA) axis abnormalities, and immune dysfunction [7–9]. The US Centers for Disease Control and Prevention (CDC) offer a clinical definition of CFS based on the Holmes and Fukuda scoring and evaluation systems [2]. However, the identification of diagnostic markers for CFS remains challenging. The discovery of such biomarkers may have been impeded by the fact that the unique biologic changes responsible for production of the original illness may no longer be present in most patients with CFS [4]. However, one consistently observed finding across patients with CFS is the exacerbation of symptoms following exercise (postexertional malaise). Therefore, the CDC symptom inventory differentiates patients with CFS from those without the syndrome according to the occurrence of postexertional malaise [10]. Thus, identification of specific biological changes associated with postexertional malaise offers a promising approach for the discovery of biomarkers of CFS.

Metabonomics focuses on all known and unknown molecular compounds rather than the analysis of individual metabolites alone, allowing new insight into the metabolic status of the entire body [11]. Specifically, an analysis of metabolite profiles before and after exercise may aid in the detection of metabolic changes associated with CFS. One of the most useful approaches for diagnosis as well as biomarker identification involves metabolite profiling of biological samples using combined gas chromatography-mass spectrometry (GC-MS) [12].

In the present study, we established a rat model of CFS using restraint-stress, forced exercise, and crowded/noisy environments. GC-MS in combination with multivariate statistical analysis was employed to evaluate changes in the metabolic profiles of urine specimens in order to identify potential biomarkers and investigate metabolic mechanisms underlying the development of CFS.

## 2. Methods

### 2.1. Establishment of the CFS Model

Twenty female Sprague-Dawley rats (weight: 200 ± 20 g, 5-6 weeks old) were obtained from the Experimental Animal Centre of Xi'an Jiaotong University (license number: SCXK (Shaan) 2012-003) in Xi'an, China. The animals were randomly allocated to either a control group or a CFS group, with 10 rats in each group. All animals were housed in a temperature-controlled environment (23 ± 2°C) with a humidity of 55% ± 15% under a 12 h light-dark cycle, with free access to water and standard rodent chow. All animal experiments were performed in accordance with the National Institutes of Health (NIH) Guidelines for the Care and Use of Laboratory Animals. The Animal Ethics Committee of Xi'an Jiaotong University (Xi'an China) reviewed and approved the entire animal protocol prior to conducting the experiments.

Based on the findings of previous reports [13–15], four methods (restraint-stress, forced exercise, and crowded and noisy environments) were adopted to mimic the multiple-factor pathogenesis of CFS. Rats in the CFS group were exposed to these conditions for 4 weeks. For restraint-stress, rats were fixed individually in a polyvinyl chloride tube (20.0 cm in length, 5.0 cm in diameter) for 4 h. For forced exercise, the rats were forced to run on a treadmill (20 m/min) for 1 h. The 10 rats in the CFS group were housed together in a standard rearing cage (crowded environment), whereas those in the control group were housed in individual cages. Rats of the CFS group were also exposed to rock music for 12 h each day (noisy environment). The protocol of the CFS model is shown in Figure S1, in Supplementary Material available online at https://doi.org/10.1155/2017/8182020.

### 2.2. Behavioral Evaluation of the CFS Model

After 4 weeks, the body weights and dietary intakes of the rats were recorded. The Morris-water-maze test, open-field test, and tail-suspension test were performed to evaluate the CFS model, as in our previous study [16]. The Morris-water-maze test was performed using a Morris-water-maze instrument (Beijing Shidi-Chuangke Co., Ltd. China). Briefly, the pool was divided into four quadrants, and a circular escape platform (12 cm in diameter) was submerged 2 cm below the surface of the water in the 2nd quadrant. The rats were trained to locate and mount the escape platform when placed in the other three quadrants. After 4 days of training, the time spent searching for the escape platform was calculated for each rat. The escape platform was then removed, and the number of times that the rats passed the previous location of the platform in 1 min was recorded.

The open-field test was conducted using an open-field behavior detector (ZH-ZFT, Huaibei Zhenghua Biological Instrument Equipment Co., Ltd., Anhui Province, China) with nine grids. An animal behavior videotracking system was used to record the locomotion of each rat. Each rat was placed in the middle grid, and the number of times that the rat crossed through the adjacent grids within 3 minutes was recorded, along with the number of times the rat stood on its hind legs. For the tail-suspension test, the tails of the rats were fixed on a horizontal board (1 m in height from the ground), and the time the rats spent motionless over a duration of 6 min was recorded.

### 2.3. Urine Sample Preparation

Urine samples were collected from all rats following behavioral evaluation (before exercise). Rats of each group were then forced to run on a treadmill (20 m/min) for 1 h, following which urine samples were again collected (after exercise). Each sample was treated with 0.1 mL of 1% sodium azide solution and stored at −80°C until measurement.

### 2.4. GC-MS Analysis of Urine Samples

The urine samples were thawed at room temperature, and 100 *μ*L of urine sample was transferred to a 1.5 mL sample vial and diluted with purified water at a ratio of 1 : 1 (v/v). Then, 10 *μ*L of urease suspension (160 mg/mL in water) was added to each sample vial, and the samples were incubated at 37°C for 1 h to decompose and remove excess urea. Subsequently, 0.35 mL of methanol was added, and the samples were mixed with 500 *μ*L of acetonitrile. The samples were then vortexed for 30 s and centrifuged for 15 min at 4°C (12,000 rpm) to remove any particulates. Next, 400 *μ*L of supernatant was transferred to a 1.5 mL sample vial, dried under nitrogen, mixed with 80 *μ*L of methoxyamine pyridine solution (20 mg/mL), and incubated at 80°C for 20 min. Next, 100 *μ*L of derivatization reagent (BSFTA + TMCS) was added, and the samples were sealed and incubated at 70°C for 1 h. Finally, 5 *μ*L of FAMEs was added to the mixed sample, and the sample was permitted to cool to room temperature, following which it was mixed thoroughly in preparation for GC-MS analysis.

GC-MS analysis was performed using an Agilent 7890 gas chromatograph system coupled with a Pegasus HT time-of-flight mass spectrometer. The system utilized an Rxi-5Sil MS column (30 m × 250 *μ*m × 0.25 *μ*m, Restek, USA). A 1 *μ*L aliquot of the analyte was injected in splitless mode. Helium was used as the carrier gas. The front inlet purge flow was 3 mL/min, and the gas flow rate through the column was 20 mL/min. The initial temperature was maintained at 50°C for 1 min and then increased to 330°C at a rate of 10°C/min, followed by maintenance at 330°C for 5 min. The injection, transfer line, and ion source temperatures were 280, 280, and 250°C, respectively. The energy was −70 eV in the electron impact mode. The mass spectrometry data were acquired in full-scan mode at an *m*/*z* range of 30–600 at a rate of 20 spectra per second after a solvent delay of 366 s.

### 2.5. Statistical Analysis

The body weights, dietary intake, and behavioral performance of the rats were analyzed using SPSS software (version 17.0). The data are presented as means ± standard deviations (SD). Differences between means were determined using independent *t*-tests. *p* values < 0.05 were considered statistically significant, while *p* values < 0.01 were considered extremely significant.

The detected peaks were aligned using manual integral methods. Peaks were analyzed only when they were consistently detected in at least 80% of the samples. All detected peaks were identified by comparing the MS spectra with those available in the Kyoto Encyclopaedia of Genes and Genomes (KEGG; http://www.genome.jp/kegg/). Only those compounds with a matching probability of greater than 70% were examined. The retention time and *m*/*z* data pairs were used as the identifiers for each peak in each sample. Statistical analysis was performed using multivariate statistics combined with univariate statistics. Normalized data were exported to SIMCA software (Version 14.0, Umetrics AB, Umea, Sweden) to perform an orthogonal partial least squares discriminant analysis (OPLS-DA), and a model was built to identify the variables that accounted for the differentiation of CFS.

## 3. Results

### 3.1. Behavioral Analysis

As shown in Table 1, significant alterations (increases or decreases) were observed in a total of seven evaluation indexes for rats in the CFS group relative to the control group, indicating that chronic stress resulted in physical and mental fatigue in the rats and that the CFS model had been successfully established.

### 3.2. GC-MS Analysis of Metabolic Profiling

**Figure 1:**
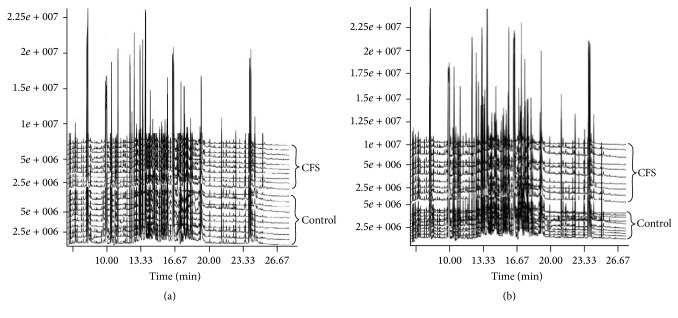
Total ion chromatograms of the urine samples obtained from the CFS and control groups. (a) Before exercise. (b) After exercise.

**Figure 2:**
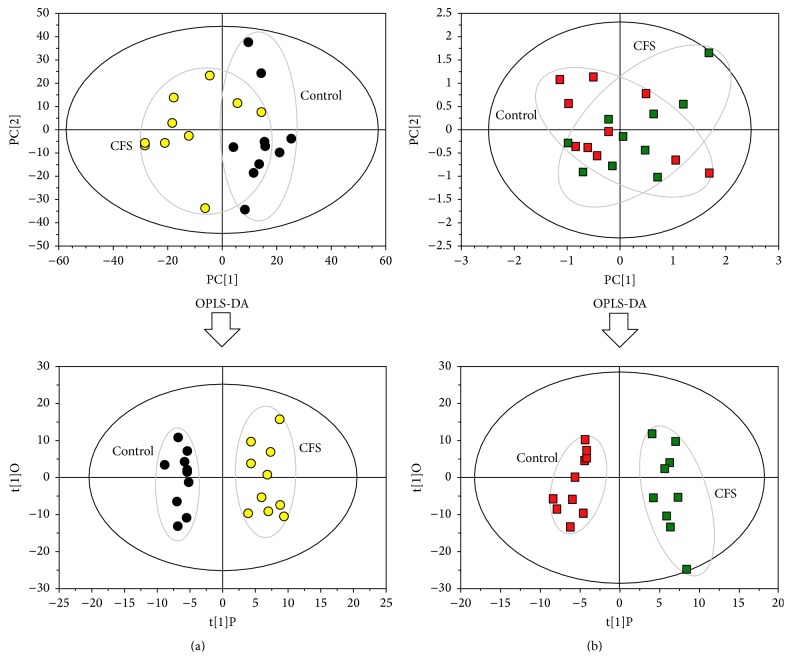
PCA scores plot and OPLS-DA scores plot of urine sample from the CFS and control groups. (a) Before exercise. (b) After exercise.

**Figure 3:**
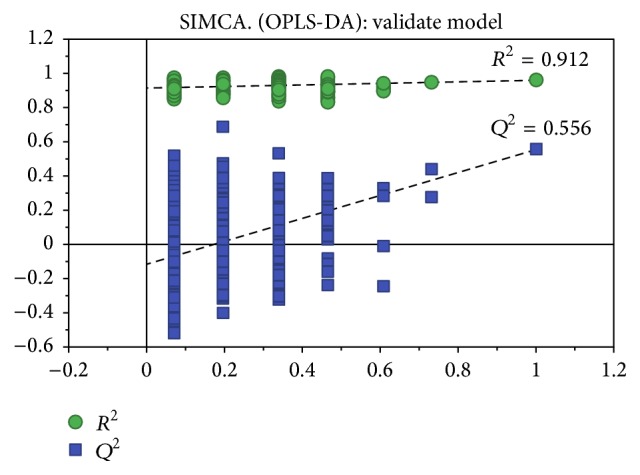
Validation of OPLS-DA model by permutation test. Validation of OPLS-DA model of rats urinary samples from two groups by permutation test (the *x*-axis means the correlation coefficient between the original *y* variable and the permutated *y* variable and the *y*-axis is the value of *R*^2^ and *Q*^2^).

The stability and repeatability of the GC-MS system for large-scale sample analysis were confirmed by analysis of pooled Quality Control (QC) samples and the retention time (RT) of the internal standard. Ten QC samples and the internal standard were analyzed for urine samples throughout the entire analysis. The principal component analysis (PCA) score plot, including all the test and QC samples, revealed that the QC sample features were tightly clustered. The relative standard deviation (RSD) of the retention times in the internal standard was less than 0.49%. Thus, the stability and repeatability of the proposed method were deemed acceptable. Typical total ion chromatograms (TICs) of urine samples from the control and CFS groups are presented in Figure 1. Subsequently, PCA was performed to obtain a comprehensive view of the metabonome, and an unsupervised multivariate data analysis was conducted to visualize the trends and outliers in the data for the control and CFS groups. As shown in Figure 2, the score plots were not clearly separated; however, the CFS group exhibited a tendency to deviate from the control group after the OPLS-DA. The model was validated using a permutation test. *R*^2^  *Y* and *Q*^2^ of the OPLS-DA model were 0.912 and 0.556 (Figure 3), respectively, indicating that the model was both reliable and predictive.

To identify the variables responsible for this large separation, the variable importance (VIP) in the projection statistics from OPLS-DA modeling and *t*-tests (*p* < 0.05) between the two groups were used to preselect variables. The GC-MS spectra of the metabolites were analyzed based on KEGG and mass spectra libraries, and metabolites were required to meet the following conditions: VIP > 1 and *p* < 0.05. As shown in Table 2, a total of 20 metabolites were identified in the CFS group before and after exercise when compared with control group. These potential biomarkers contributed to the discrimination of the metabolic profiles between the CFS and control groups.

### 3.3. Pathway Topology Analysis

**Figure 4:**
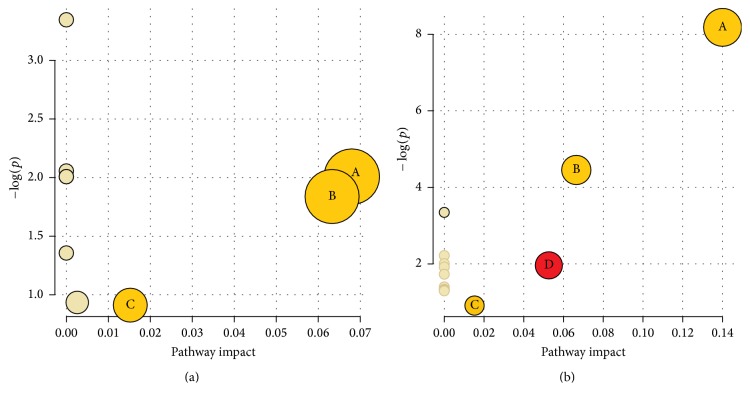
Results of the metabolic pathway topology analysis. (a) Before exercise. (b) After exercise. (A) TCA cycle. (B) Alanine, aspartate, and glutamate metabolism. (C) Steroid hormone biosynthesis. (D) Sphingolipid metabolism.

To identify possible metabolic pathways affected by CFS, the 20 identified metabolites were analyzed using MetPA (http://www.metaboanalyst.ca/). As shown in Table 3, nine (preexercise) and 14 (postexercise) metabolic pathways were generated in CFS group in comparison with the control groups, and the pathway impact values of those metabolic pathways were calculated via a pathway topology analysis with a threshold of 0.01. The results revealed that three metabolic pathways (TCA cycle; alanine, aspartate, and glutamate metabolism; and steroid hormone biosynthesis) of the CFS rats were filtered out before and after exercise, while sphingolipid metabolism alone exhibited significant alterations after exercise only (Figure 4).

**Figure 5:**
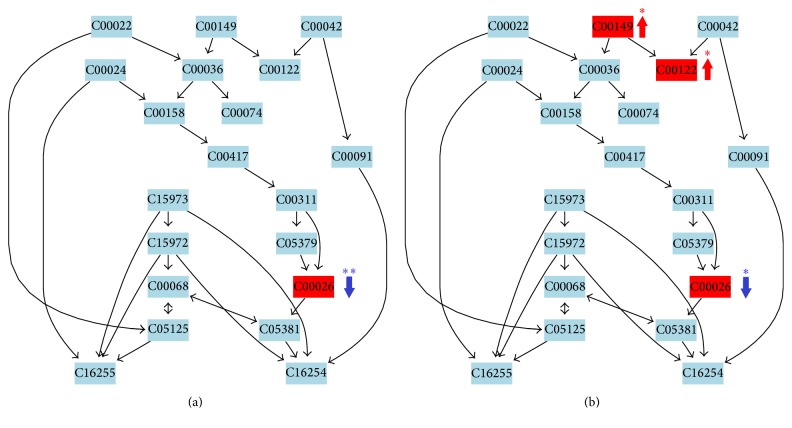
Metabolic pathway of TCA cycle. (a) Before exercise. Key metabolite: *α*-ketoglutaric acid (C00026). (b) After exercise. Key metabolites: malic acid (C00149), fumaric acid (C00122), and *α*-ketoglutaric acid (C00026). ↑: upregulated, ↓: downregulated, ^*∗*^*p* < 0.05, and ^*∗∗*^*p* < 0.01 versus control.

**Figure 6:**
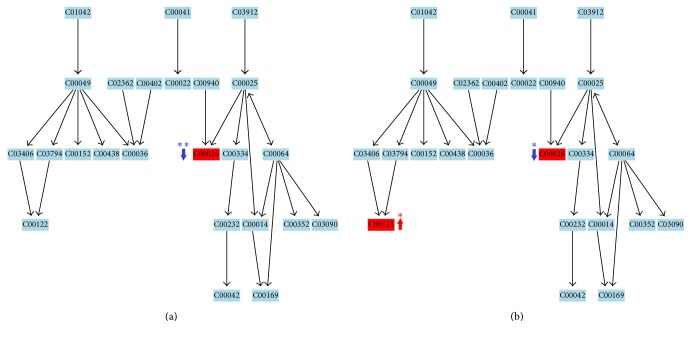
Metabolic pathway of alanine, aspartate, and glutamate metabolism. (a) Before exercise. Key metabolite: *α*-ketoglutaric acid (C00026). (b) After exercise. Key metabolites: fumaric acid (C00122) and *α*-ketoglutaric acid (C00026). ↑: upregulated, ↓: downregulated, ^*∗*^*p* < 0.05, and ^*∗∗*^*p* < 0.01 versus control.

**Figure 7:**
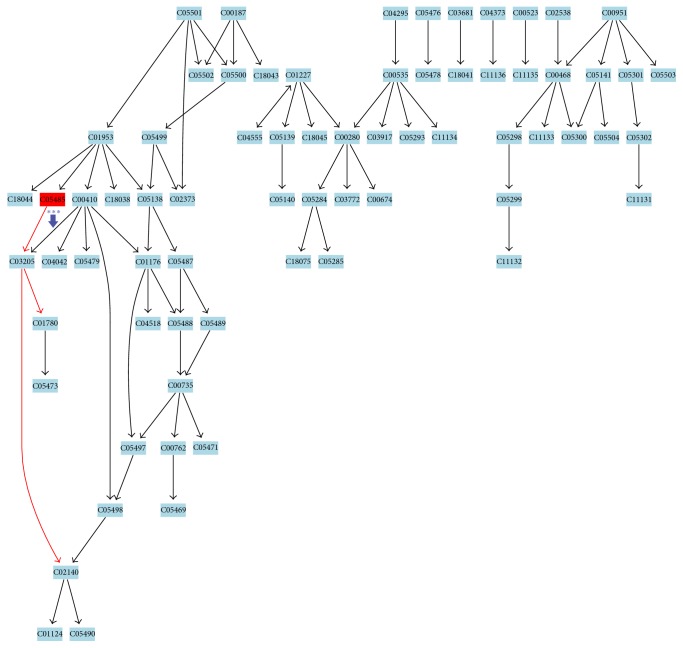
Metabolic pathway of steroid hormone biosynthesis. Related metabolites: 21-hydroxypregnenolone (C05485), deoxycorticosterone (C03205), corticosterone (C02140), and aldosterone (C01780). ↓: downregulated and ^*∗∗∗*^*p* < 0.001 versus control.

**Figure 8:**
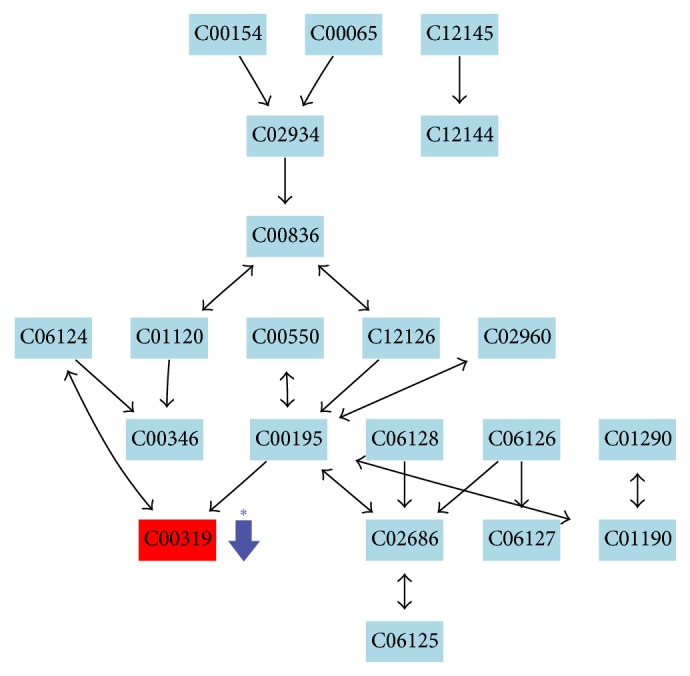
Metabolic pathway of sphingolipid metabolism. Key metabolites: sphingosine (C00319). ↓: downregulated and ^*∗*^*p* < 0.05 versus control.

**Figure 9:**
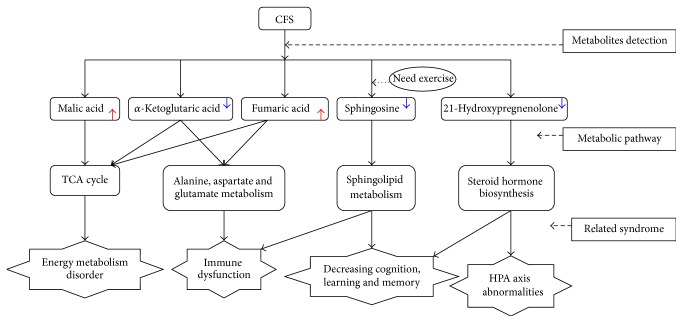
Metabolic mechanisms of CFS.

Metabolites detected before and after exercise differed in the metabolic pathways associated with the TCA cycle. As shown in Figure 5 and Table 2, *α*-ketoglutaric acid was markedly decreased both before exercise (*p* < 0.01) and after exercise (*p* < 0.05), whereas fumaric acid and malic acid were increased after exercise (*p* < 0.05). In metabolic pathways associated with alanine, aspartate, and glutamate metabolism, similar changes in *α*-ketoglutaric acid were observed before and after exercise, while levels of fumaric acid decreased after exercise (Figure 6). Our findings revealed that the level of 21-hydroxypregnenolone in CFS rats was significantly decreased (*p* < 0.001) in the pre- and postexercise conditions in comparison with the controls (Table 2). As shown in Figure 7, corticosterone and aldosterone were the direct and indirect downstream products based on the analysis of 21-hydroxypregnenolone metabolic pathway. In addition, increases in sphingosine were detected (*p* < 0.05) only after exercise (Figure 8). Therefore, we summarized the metabolic mechanisms of CFS according to the above results (shown in Figure 9).

## 4. Discussion

In the present study, we established a rat model of CFS over 4 weeks using four types of stimulation: restraint-stress, forced exercise, crowded environment, and noisy environment [17]. As patients with CFS typically present with behavioral symptoms such as memory deficits, inattention, disinterest in new activities, and depression [18–20], we utilized a variety of behavioral tests to assess the validity of our model. The Morris-water-maze test is often used to detect the learning and memory capabilities of animals [21]: rodents will strive to escape from water and will exploit spatial memory to exit the water as quickly as possible. The open-field test evaluates the exploration activities of the animals and their emotions in a new environment and can be used to examine their anxiety and excitability, while the tail-suspension tests reflect disappointment and depression in animals. Our results indicate that chronic stress resulted in physical and mental fatigue in the rats and that the methods used to generate the CFS model were successful. The results of our pathway topology revealed the dysfunction of the TCA cycle; alanine, aspartate, and glutamate metabolism; and steroid hormone biosynthesis in CFS rats.

The persistent fatigue and muscle weakness were the symptoms in CDC symptom inventory list that differentiated subjects with CFS from those without the syndrome [22, 23]. McCully and Natelson reported that the lactic acid level increased and the oxygen transfer ability decreased in CFS patients after exercise [24]. The blood rheology research showed there was the significant microcirculation disturbance in CFS patients, which affected not only the movement of red blood cells in capillaries but also the metabolism of oxygen and lactate [25]. The disorder of the aerobic metabolism caused the lack of the energy supply in muscle contraction, which leads to body fatigue. There was close relationship between the energy metabolism and the fatigue or muscle weakness. The TCA cycle represents the intersection of the catabolism of sugar, fat, and amino acids. The intermediate metabolites of the TCA cycle are also the origins of many biosynthetic pathways. Therefore, TCA status is a comprehensive reflection of energy metabolism in the body [26]. So we concluded that dysfunction in TCA cycle metabolism could explain those fatigue symptoms in CFS.

Additionally, a number of studies have demonstrated a state of generalized immune activation and selective immune dysfunction, in patients with CFS [27, 28]. Other authors also reported that the lymphocyte proliferation function and monocytes phagocytosis decreased in CFS patients, as well as number and activity of natural killer (NK) cell [29, 30]. The process of chronic fatigue stimulations accelerated leukocyte apoptosis and upset the balance of the immunity system in the regulation of cell metabolism [31]. In metabolites analysis, *α*-ketoglutaric acid and fumaric acid were filtered (before exercise and/or after exercise) which associated with not only TCA cycle metabolism but also alanine, aspartate, and glutamate metabolism. It is well known that the alanine, aspartate, and glutamate metabolism play transfer amidogen role in amino acid metabolism, which directly or indirectly affects the formation of antibody and glutamine. The latter can improve the proliferation and differentiation of lymphocytes [32]. So we concluded that the immunity dysfunction of CFS patients associated with metabolic disturbance in the alanine, aspartate, and glutamate metabolism.

Besides the reduced learning ability, short-term memory loss, and difficulty concentrating, the cognitive biases on diseases also belong to CFS identification standard of CDC. Those CFS patients with cognitive biases think that disease is an unpredictable and uncontrollable physiological problem and then actually increase their asthenic feeling and fatigue feeling [33, 34]. Some studies have demonstrated the potential involvement of the central and autonomic nervous system in patients with CFS [35, 36]. In the present research, the results revealed a visible perturbation of the urine metabolic profiles in CFS rats, with steroid hormone biosynthesis identified as the key metabolic pathway associated with the mechanism underlying CFS. It is well known that 21-hydroxypregnenolone plays an important role during steroid hormone biosynthesis and can improve memory, prevent fatigue, and relieve stress [37, 38]. HPA axis abnormalities have also been proposed as potential modes of CFS pathogenesis [8]. Some research has indicated that cortisol levels in patients with CFS are lower than those of healthy controls due to damage to cortical hormone markers [39]. Therefore, we inferred that 21-hydroxypregnenolone may be a biomarker of HPA axis abnormalities in patients with CFS.

Our results further suggest that sphingolipid metabolism is significantly associated with the effect of exercise on metabolic pathways in patients with CFS. It is generally known that sphingolipid is one important component of the cell membrane. And ceramide, sphingosine, and sphingosine-1-phospate, named “sphingomyelin rheostat,” are the most important metabolites in sphingolipid metabolism. Some research has indicated that the unbalance of “sphingomyelin rheostat” caused the memory loss and cognitive decline in patients of Alzheimer's disease and autism [40, 41]. Sphingosine can participate in the generation of ceramide during sphingomyelin metabolism and promote immune function, cognition, and learning and memory in rats [42, 43].

The intensive exercise can aggravate the symptoms of CFS patients [44], and then we can obtain the more physiological information from them after exercise. Our results showed that the sphingosine levels significantly decreased in CFS rats after exercise. Therefore, we regarded sphingosine as the key biomarker underlying the effects of exercise which can explains the immune dysfunction and the decline of cognition, learning, and memory (as shown in Figure 9). However, the mechanism needs further to be explored on the reduced sphingosine level because of exercise.

## 5. Conclusions

In the present study, we employed a metabonomics approach to investigate the potential underlying mechanisms of CFS. Based on the multivariate statistical analysis, three metabolic pathways and a total of six metabolites were identified in comparison with the control group. Significant alterations were specifically observed in the sphingolipid pathway following exercise. Our findings indicate that sphingosine levels following exercise may represent a novel biomarker of CFS. However, patients with CFS differ to some extent from mere animal models of the disease. Therefore, further studies involving patients with CFS are required in order to uncover additional metabonomic information regarding the development of the condition.

## Supplementary Material

Figure S1: The protocol of the CFS model. Four methods (restraint-stress, forced exercise, and crowded and noisy environments) were adopted to mimic the multiple-factor pathogenesis of CFS.

## Figures and Tables

**Table 1:** Results of the behavioral tests.

Groups (*n* = 10)	Body weight (g)	Diet		Morris-water-maze test		Open-field test		Tail-suspension test
		Fodder (g/d)	Water (mL/d)	Time of searching for platform(s)	Number of times	Number of times	Number of standing events	Motionless time(s)
Control	201.37 ± 9.09	20.84 ± 4.45	54.65 ± 8.97	31.22 ± 14.53	1.38 ± 1.30	46.25 ± 5.23	15.88 ± 4.12	78.75 ± 15.37
CFS	177.90 ± 11.13^*∗∗*^	16.61 ± 3.15^*∗∗*^	45.89 ± 12.45	50.09 ± 18.09^*∗*^	0.75 ± 0.71	33.69 ± 5.19^*∗∗*^	11.50 ± 2.48^*∗*^	100.50 ± 11.94^*∗∗*^

^*∗*^ *P* < 0.05 and ^*∗∗*^*P* < 0.01 versus the control group.

**Table 2:** Potential biomarkers and the related pathways in CFS group in comparison with the control group.

	Number	RT	*m/z*	KEGG	Formula	Metabolite	Trend^a^	Related pathway
Pre-exercise	1	8.87	188	C00383	CH_2_(COOC_2_H_5_)_2_	Malonic acid	↓^*∗*^	Pyrimidine metabolism; *β*-alanine metabolism
	2	13.64	247	C01013	C_3_H_6_O_3_	3-Hydroxypropionic acid	↓^*∗*^	Pyrimidine metabolism; *β*-alanine metabolism; propanoate metabolism
	3	13.84	304	C00026	C_5_H_7_NO_5_	*α*-Ketoglutaric acid	↓^*∗∗*^	TCA cycle; alanine, aspartate, and glutamate metabolism
	4	14.21	164	C05593	C_8_H_7_O_3_	3-Hydroxyphenylacetic acid	↓^*∗∗*^	Tyrosine metabolism; phenylalanine metabolism
	5	14.46	267	C00156	C_7_H_6_O_3_	4-Hydroxybenzoic acid	↓^*∗∗*^	Phenylalanine metabolism
	6	18.01	202	C00954	C_10_H_9_NO_2_	Indole-3-acetic acid	↓^*∗*^	Tryptophan metabolism
	7	18.24	319	C00880	C_6_H_10_O_7_	Galactonic acid	↓^*∗*^	Galactose metabolism
	8	18.27	333	C00257	C_6_H_12_O_7_	Gluconic acid	↓^*∗∗*^	Pentose phosphate pathway; biosynthesis of antibiotics
	9	23.19	230	C00294	C_10_H_12_N_4_O_5_	Inosine	↓^*∗*^	Purine metabolism
	10	26.58	73	C05485	C_6_H_11_NO_3_	21-Hydroxypregnenolone	↓^*∗∗∗*^	Steroid hormone biosynthesis

Post-exercise	1	8.87	188	C00383	CH_2_(COOC_2_H_5_)_2_	Malonic acid	↓^*∗∗*^	Pyrimidine metabolism; *β*-alanine metabolism
	2	11.00	245	C00122	C_4_H_4_O_4_	Fumaric acid	↑^*∗*^	TCA cycle; alanine, aspartate, and glutamate metabolism
	3	11.65	184	C01732	C_5_H_6_O_4_	2-Methylfumarate	↑^*∗*^	C_5_-branched dibasic acid metabolism
	4	12.73	233	C00149	C_4_H_6_O_5_	Malic acid	↑^*∗*^	TCA cycle
	5	13.83	304	C00026	C_5_H_7_NO_5_	*α*-Ketoglutaric acid	↓^*∗*^	TCA cycle; alanine, aspartate, and glutamate metabolism
	6	14.48	292	C00898	C_4_H_6_O_6_	Tartaric acid	↓^*∗∗*^	TCA cycle
	7	18.27	333	C00257	C_6_H_12_O_7_	Gluconic acid	↓^*∗*^	Pentose phosphate pathway; biosynthesis of antibiotics
	8	18.77	117	C00249	C_16_H_32_O_2_	Palmitic acid	↑^*∗*^	Fatty acid metabolism; biosynthesis of unsaturated fatty acids
	9	22.16	204	C00319	C_14_H_31_NO_6_S	Sphingosine	↓^*∗*^	Sphingolipid metabolism
	10	26.57	73	C05485	C_6_H_11_NO_3_	21-Hydroxypregnenolone	↓^*∗∗∗*^	Steroid hormone biosynthesis

^a^(↑): upregulated; (↓): downregulated. ^*∗*^*p* < 0.05; ^*∗∗*^*p* < 0.01; ^*∗∗∗*^*p* < 0.001.

**Table 3:** Impact scores for metabolic pathways in CFS.

Time	Pathway Name	Total	Hits	*p*	−log⁡(*p*)	Holm *p*	FDR	Impact score	Ranking list^*∗*^
Before exercise	TCA cycle	20	1	0.134	2.008	1.00	1.00	0.068	1
	Alanine, aspartate and glutamate metabolism	24	1	0.159	1.839	1.00	1.00	0.063	2
	Steroid hormone biosynthesis	70	1	0.402	0.912	1.00	1.00	0.015	3
	Purine metabolism	68	1	0.393	0.935	1.00	1.00	0.003	—
	D-Glutamine and D-glutamate metabolism	5	1	0.035	3.347	1.00	1.00	0.000	—
	beta-Alanine metabolism	19	1	0.128	2.056	1.00	1.00	0.000	—
	Propanoate metabolism	20	1	0.134	2.008	1.00	1.00	0.000	—
	Butanoate metabolism	20	1	0.134	2.008	1.00	1.00	0.000	—
	Tryptophan metabolism	41	1	0.258	1.357	1.00	1.00	0.000	—

After exercise	TCA cycle	20	3	0.000	8.181	0.02	0.02	0.140	1
	Alanine, aspartate, and glutamate metabolism	24	2	0.012	4.454	0.93	0.47	0.067	2
	Sphingolipid metabolism	21	1	0.141	1.963	1.00	1.00	0.053	3
	Steroid hormone biosynthesis	70	1	0.402	0.912	1.00	1.00	0.015	4
	D-Glutamine and D-glutamate metabolism	5	1	0.035	3.347	1.00	0.95	0.000	—
	Glyoxylate and dicarboxylate metabolism	16	1	0.109	2.219	1.00	1.00	0.000	—
	Butanoate metabolism	20	1	0.134	2.008	1.00	1.00	0.000	—
	Pyruvate metabolism	22	1	0.147	1.919	1.00	1.00	0.000	—
	Fatty acid elongation in mitochondria	27	1	0.177	1.730	1.00	1.00	0.000	—
	Fatty acid metabolism	39	1	0.247	1.400	1.00	1.00	0.000	—
	Tyrosine metabolism	42	1	0.263	1.336	1.00	1.00	0.000	—
	Biosynthesis of unsaturated fatty acids	42	1	0.263	1.336	1.00	1.00	0.000	—
	Fatty acid biosynthesis	43	1	0.268	1.315	1.00	1.00	0.000	—
	Arginine and proline metabolism	44	1	0.274	1.295	1.00	1.00	0.000	—

^*∗*^Threshold of the impact score >0.01.
