# Supplementary figures and images for: Detection of Urine Metabolites in a Rat Model of Chronic Fatigue Syndrome before and after Exercise
Source: Biomed Res Int. 2017 Mar 22;2017:8182020. doi: 10.1155/2017/8182020 (PMC5380834; doi:10.1155/2017/8182020)

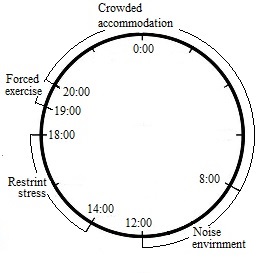


Figure S1. The protocol of the CFS model

Supplement: Supplementary file 1 — Figure S1: The protocol of the CFS model. Four methods (restraint-stress, forced exercise, and crowded and noisy environments) were adopted to mimic the multiple-factor pathogenesis of CFS. [file 8182020.f1.docx]
